# The Application of the Ten Group Classification System (TGCS) in Caesarean Delivery Case Mix Adjustment. A Multicenter Prospective Study

**DOI:** 10.1371/journal.pone.0062364

**Published:** 2013-06-05

**Authors:** Gianpaolo Maso, Salvatore Alberico, Lorenzo Monasta, Luca Ronfani, Marcella Montico, Caterina Businelli, Valentina Soini, Monica Piccoli, Carmine Gigli, Daniele Domini, Claudio Fiscella, Sara Casarsa, Carlo Zompicchiatti, Michela De Agostinis, Attilio D'Atri, Raffaela Mugittu, Santo La Valle, Cristina Di Leonardo, Valter Adamo, Silvia Smiroldo, Giovanni Del Frate, Monica Olivuzzi, Silvio Giove, Maria Parente, Daniele Bassini, Simona Melazzini, Secondo Guaschino, Francesco De Seta, Sergio Demarini, Laura Travan, Diego Marchesoni, Alberto Rossi, Giorgio Simon, Sandro Zicari, Giorgio Tamburlini

**Affiliations:** 1 Department of Obstetrics and Gynaecology, Institute for Maternal and Child Health, IRCCS Burlo Garofolo, Trieste, Italy; 2 Epidemiology and Biostatistics Unit, Institute for Maternal and Child Health, IRCCS Burlo Garofolo, Trieste, Italy; 3 Department of Obstetrics and Gynaecology, Hospital of Gorizia, Gorizia, Italy; 4 Department of Obstetrics and Gynaecology, Hospital of Latisana, Latisana, Italy; 5 Department of Obstetrics and Gynaecology, Hospital of Palmanova, Palmanova, Italy; 6 Department of Obstetrics and Gynaecology, Hospital of Monfalcone, Monfalcone, Italy; 7 Department of Obstetrics and Gynaecology, San Giorgio Private Hospital, Pordenone, Italy; 8 Department of Obstetrics and Gynaecology, S. Maria degli Angeli Hospital, Pordenone, Italy; 9 Department of Obstetrics and Gynaecology, Hospital of San Daniele del Friuli, San Daniele del Friuli, Italy; 10 Department of Obstetrics and Gynaecology, S.Maria dei Battuti Hospital, San Vito al Tagliamento, Italy; 11 Department of Obstetrics and Gynaecology, S.Antonio Abate Hospital, Tolmezzo, Italy; 12 Department of Neonatal Intensive Care Unit, Institute for Maternal and Child Health, IRCCS Burlo Garofolo, Trieste, Italy; 13 Department of Obstetrics and Gynaecology, University of Udine, Udine, Italy; 14 Regional Health Agency, Friuli Venezia Giulia, Italy; 15 Department of Epidemiology and Statistics, University La Sapienza, Rome, Italy; 16 Scientific Committee, Institute for Maternal and Child Health, IRCCS Burlo Garofolo, Trieste, Italy; 17 Multicenter Study Group on mode of delivery in Friuli Venezia Giulia, Italy; Italian National Agency for Regional health Services, Italy

## Abstract

**Background:**

Caesarean delivery (CD) rates are commonly used as an indicator of quality in obstetric care and risk adjustment evaluation is recommended to assess inter-institutional variations. The aim of this study was to evaluate whether the Ten Group classification system (TGCS) can be used in case-mix adjustment.

**Methods:**

Standardized data on 15,255 deliveries from 11 different regional centers were prospectively collected. Crude Risk Ratios of CDs were calculated for each center. Two multiple logistic regression models were herein considered by using: Model 1- maternal (age, Body Mass Index), obstetric variables (gestational age, fetal presentation, single or multiple, previous scar, parity, neonatal birth weight) and presence of risk factors; Model 2- TGCS either with or without maternal characteristics and presence of risk factors. Receiver Operating Characteristic (ROC) curves of the multivariate logistic regression analyses were used to assess the diagnostic accuracy of each model. The null hypothesis that Areas under ROC Curve (AUC) were not different from each other was verified with a Chi Square test and post hoc pairwise comparisons by using a Bonferroni correction.

**Results:**

Crude evaluation of CD rates showed all centers had significantly higher Risk Ratios than the referent. Both multiple logistic regression models reduced these variations. However the two methods ranked institutions differently: model 1 and model 2 (adjusted for TGCS) identified respectively nine and eight centers with significantly higher CD rates than the referent with slightly different AUCs (0.8758 and 0.8929 respectively). In the adjusted model for TGCS and maternal characteristics/presence of risk factors, three centers had CD rates similar to the referent with the best AUC (0.9024).

**Conclusions:**

The TGCS might be considered as a reliable variable to adjust CD rates. The addition of maternal characteristics and risk factors to TGCS substantially increase the predictive discrimination of the risk adjusted model.

## Introduction

The worldwide rise in caesarean delivery (CD) rates is becoming a major public health issue and cause of debate due to the concern that high CD rates are not associated with an improvement of perinatal mortality and may increase maternal risks [1], [2].

At present, the cesarean delivery rate in Italy is one of the highest in the world: at the end of ’70, it was around 11% and subsequently showed a progressive increase, reaching percentages around 39% in 2008, with significant inter-regional variations (from 24.1% to 61.8%) [1], [3]. Many potential causes have been advocated to justify this phenomenon: the wrong assumption that cesarean section is safer than vaginal delivery, socio-cultural attitudes [4], advanced maternal age [5], obesity [6], maternal request [7], and finally the attitude of obstetricians to a defensive medicine for the fear of medico-legal litigations [8].

In 1985 the World Health Organization (WHO) stated that caesarean section rates higher than 10–15% are not justified and the rise of caesarean deliveries led inevitably to a worldwide interest and debate, on both the causes and the appropriateness of this increase [9], [10]. However, the ideal cut-off proposed by WHO has been criticized considering that CD rates might differ among institutions because of different organizational settings, obstetric populations, local resources and available expertise: it is clear that centers handling more deliveries presenting risk factors, such as multiple gestations, preterm deliveries or pre-existing medical conditions, cannot be expected to uphold the same CD rates as observed in centers with higher proportion of uncomplicated pregnancies [11].

It is well known that the CD rate is used as an important indicator of obstetric quality, with the implicit assumption that low rates may reflect efficient and appropriate care. Although crude CD rates are often used in such profiles, many studies demonstrated poor agreement between unadjusted and adjusted institutional rates: if institutional CD rates are compared without adjusting for differences in the patients' population, hospitals serving high-risk populations will have high rates and will appear to dispense poor care, even if they are providing top quality care [12]–[16].

Risk adjustment of CD rates overcomes the problem of patient variation between hospitals, leaving residual differences being explained by differences in decision-making between institutions [17].

Antenatal risk factors and maternal characteristics are commonly used as customary variables to evaluate inter-institutional variations of adjusted CD rates [18]. Recently, Colais et al. proposed to introduce the 10-group classification system (TGCS) in risk adjustment, considering this system as the best method for categorizing the mode of delivery and fulfilling the criteria of mutually exclusive and totally inclusive collection [11], [19]. They concluded that risk adjusted evaluation of CD rates should be done combining TGCS and obstetric risk factors [20]. However, their study did not assess the reliability of this model for CD case-mix adjustment.

The aim of our study, carried out on more than 15 thousand deliveries from all of the 11 obstetric departments of our Region, was to evaluate whether the TGCS can be used in case-mix CD rate adjustment. To assess this objective, we compared different models, considering in multiple logistic regression analyses, either the maternal – obstetric variables and risk factors as customary variables or the TGCS with or without the association of maternal characteristic and risk factors.

## Methods

An 18-months prospective study collected data on mode of delivery from all births of the 11 single-institutional obstetric cohorts of Friuli Venezia Giulia (range 369–1,810 deliveries/year/unit). Friuli Venezia Giulia is a region of North-Eastern Italy accounting roughly 10,000 deliveries per year with one of the lowest overall regional CD rates in Italy (23.4% in 2010). The source institutions, referred as institutions A to M, are first level departments serving low risk pregnancies, except for centers I and M working for a mixed population with the availability of a Neonatal Intensive Care Unit (NICU, third referral units).

The units differed for number of deliveries/year as follow: units A, B, C, E, F, G, H, and L had less than 1,000 deliveries/year; center D accounted for 1,000–1,500 deliveries/year; 1,500–2,000 deliveries/year were assisted in institutions M and L.

To avoid potential information bias due to different definitions on collected data, we created a regional standardized computerized database with the collaboration of all centers. Data on institutional deliveries were prospectively collected at the time of delivery and were systematically reviewed every month by the referent obstetrician of each center. Special attention was devoted to overall data completeness and accuracy and during the study period two of the authors (GM and SA) organized periodical multicenter meetings to discuss the results and provide assistance. All women provided written informed consent to include their records in the presentation of summary data for births.

The study was approved by the institutional review board of the coordinating center (Technical Scientific Committee- CTS-, Institute for Maternal and Child Health – IRCCS Burlo Garofolo, Trieste, project 86/05 – February, the 28^th^, 2007) and access to the data was approved by all hospital trust administrations. According to Italian law on privacy (Art. 20–21, DL 196/2003), data were anonymized at every institution where each patient was assigned a unique identifier. This identifier did not allow to trace the patient's identity and other sensitive data.

To assess the inter-institutional differences in mode of delivery, defined as vaginal or caesarean, maternal and pregnancy-related characteristics, risk factors known to increase the likelihood of CD and the TGCS were considered. Maternal characteristics included maternal age (<20, 20–24, 25–29, 30–35, >35 years) and pre-pregnancy body mass index (BMI), classified as underweight (less than 18), normal (between 18 and 25), overweight (between 26 and 30) and obese (over 30).

Parity (nulliparous, multiparous), past CD (none, one, two or more), gestational age at delivery (<30, 30–36, 37–41, >41 weeks), fetal lie or presentation (cephalic, abnormal lie), pregnancy with multiples and infant birth weight (<1000, 1000–1500, 1500–2500, 2500–4000, >4000 grams) were considered as pregnancy-related variables.

Pregnancy was classified as at low, intermediate or high risk on the basis of the following definitions: low risk if no pre-existing or ante partum risk factors were identified; intermediate risk in presence of pre-existing maternal medical conditions complicating pregnancy, but not representing per se an absolute indication for CD or induction of labor (i.e. polyhydramnios, chronic hypertension, pregnancy-associated hypertension, gestational diabetes, presence of multiple myomas, obstetric cholestasis and Rh-isoimmunization); high risk if pre-existing maternal diseases or other obstetric conditions were present and suggesting the termination of pregnancy by CD or induction of labor (such as HIV infection, past myomectomy, pre-existing diabetes, severe pre-eclampsia, placenta previa, severe oligohydramnios and severe intrauterine growth restriction) [18].

The study population was also assessed using the TGCS (Table 1) with specific reference to the size and CD rates in each group. Groups 6, 7 and 9 were merged in a single group (all abnormal lie/presentation) because of the overall extremely high risk of CD in these groups. In each group the indications for induction of labor and CD were reported according to standardized definitions ( Tables S1–S2). For cases in which more than one indication was present, the obstetrician was asked to report the main indication for induction or CD. Only cases with complete data on all of the above indicated variables were considered for the analysis.

**Table 1:** The 10-group classification.

Group	Classification
1	Nulliparous, single cephalic, ≥37 weeks, in spontaneous labor
2	Nulliparous, single cephalic, ≥37 weeks induced labor or pre-labor CD
3	Multiparous (excluding previous CD), single cephalic, ≥37 weeks, in spontaneous labor
4	Multiparous (excluding previous CD), single cephalic, ≥37 weeks, induced labor or pre-labor CD
5	Previous CD, single cephalic, ≥37 weeks
6	All nulliparous breeches
7	All multiparous breeches (including previous CD)
8	All multiple pregnancies (including previous CD)
9	All transverse/oblique lies (including previous CD)
10	All preterm single cephalic, <37 weeks, including previous CD

CD, caesarean delivery.

Statistical analysis was carried out to calculate firstly the unadjusted rates and Risk Ratios (crude RRs) of delivery by caesarean section for each center. Secondly, different multiple logistic regression models (adjusted ORs were converted to adjusted RRs) [21] were used to estimate the probability of CD. In model 1, CD rates were adjusted considering maternal characteristics, pregnancy related variables and antenatal risk classification, as previously described. Model 2 was instead based on the TGCS. This adjustment was carried out considering the ten obstetric groups either without or including maternal characteristics and risk factors (models 2a and 2b respectively). In all of the analyses, the referent center was considered the institution with the lowest CD rate. Statistical analysis was carried out with Stata/IC, version 11.2 for Windows [22] and results were expressed as Risk Ratios (RRs) and 95% Confidence Intervals (95% CI), considering p<0.05 as statistically significant.

Receiver Operating Characteristic (ROC) curves of the multivariate logistic regression analyses were used to compare the models. The null hypothesis that Areas under the ROC Curve (AUC) were not different from each other was verified with a Chi Square test and post hoc pairwise comparisons were carried out using a Bonferroni correction.

## Results

**Figure 1:**
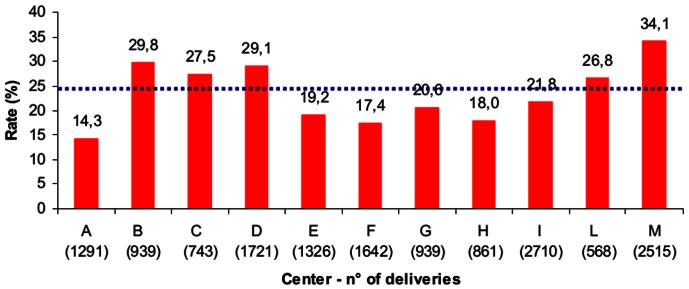
Inter-institutional caesarean delivery rates: data are presented as percentages (number of caesarean deliveries/total number of deliveries. The dot line represents the average of overall caesarean delivery rates.

In the study period, information was collected on a total of 15,727 deliveries. Because of missing data on maternal age or BMI, 472 cases were excluded from the analysis (3%), resulting in a study population of 15,255 deliveries. Distributions of non-missing independent variables and CD rates were similar across the analyzed and excluded samples. In the final cohort, the average of overall CD rates was 24.0% (3,652/15,255), ranging from 14.3% to 34.1% (Figure 1). Overall mean maternal age and pre-pregnancy BMI were respectively 31.7 years (standard deviation – SD 5.2) and 22.6 (SD 3.8). The overall mean of gestational age at delivery was 39.0 weeks (SD 1.8). Multiple pregnancies were 230 (1.5%) and nulliparity accounted for 54% of pregnancies (8,236/15,255). Previous CDs and other risk factors, as stated in the methods section, were respectively present in 9.0% (1,382/15,255) and 13.4% (2,058/15,255) of all cases.

As for the relative size of the ten groups, the most represented groups were respectively group 1 (36.1%–5,516/15,255), group 3 (29.3%–4,474/15,255), group 2 (11.2%–1,703/15,255) and group 5 (7.8%–1,199/15,255).

### Bivariate analysis

CD rates differed among institutions and all centers had crude RRs significantly higher than center A, considered as the referent (CD rate 14.3% – Figure 1, Table 2). Regarding the association between maternal characteristics and/or pregnancy related variables and mode of delivery, we observed that CD rates were significantly higher in mothers between 30 and 35 years of age (23.6%) or over 35 (30.6%), if compared with women less than 30 years old. The CD rate differed significantly among BMI classes: CD occurred more frequently in overweight and obese women (32.4% and 37.9% respectively) than in cases presenting normal (22.5%) or low BMI (19.4%). As for gestational age, CD was significantly associated with preterm delivery, whereas CD rates did not differ significantly between cases beyond 41 weeks and pregnancies at term (37–41 weeks). In bivariate analysis, the CD rate did not vary significantly between nulliparous and multiparous women. Pregnancy labeled as at intermediate or high risk showed to have a 1.8 and 2.5-fold increased risk of caesarean section respectively. As expected, abnormal lie (breech or transverse), one or more previous CDs and multiple pregnancies represented the most significant conditions associated with CD. Regarding the ten groups, all groups, except multiparous, cephalic presentation, at term, without scar and in spontaneous labor, had a significant higher risk of CD if compared to the referent group of nulliparous at term, cephalic presentation in spontaneous labor (group 1).

**Table 2:** Mode of delivery (vaginal vs. caesarean) according to maternal characteristics, obstetric variables and 10-Group classification.

	Mode of delivery		Caesarean Delivery	
Variable	Vaginal, n (%)	Caesarean, n (%)	Crude RR [95% CI]	p-Value
Maternal Age (years)				
20–24	1,052/1,279 (82.2)	227/1,279 (17.8)	Referent	Referent
<20	153/182 (84.1)	29/182 (15.9)	0.91 [0.64–1.24]	0.558
25–29	2661/3,327 (80.0)	666/3,327 (20.0)	1.12 [0.99–1.26]	0.076
30–35	5148/6,738 (76.4)	1590/6,738 (23.6)	1.30 [1.16–1.44]	0.000
>35	2589/3,731 (69.4)	1140/3,729 (30.6)	1.63 [1.48–1.79]	0.000

BMI				
18.5–25	9,219/11,895 (77.5)	2676/11,895 (22.5)	Referent	Referent
<18.5	901/1,117 (80.7)	216/1,117 (19.3)	0.86 [0.76–0.97]	0.018
26–30	1,101/1,628 (67.6)	5,27/1,628(32.4)	1.43 [1.33–1.54]	0.000
>30	382/615 (62.1)	233/615 (37.9)	1.66 [1.50–1.83]	0.000

Gestational Age (weeks)				
37–41	10,380/13,129 (79.1)	2,749/13,129 (20.9)	Referent	Referent
<30	17/74 (23.0)	57/74 (77.0)	3.37 [2.94–3.68]	0.000
30–36	1057/1,859 (56.9)	802/1,859 (43.1)	1.99 [1.88–2.09]	0.000
>41	149/193 (77.2)	44/193 (22.8)	1.08 [0.83–1.38]	0.529

Risk factors				
No risk	10,499/13,197 (79.6)	2,688/13,197 (20.4)	Referent	Referent
Intermediate risk	650/1,058 (61.4)	408/1,058 (38.6)	1.84 [1.70–1.98]	0.000
High risk	454/1,000 (45.4)	546/1,000 (54.5)	2.54 [2.40–2.68]	0.000

Parity				
Multiparous	5,354/7,020 (76.3)	1,666/7,020 (23.7)	Referent	Referent
Nulliparous	6,249/8,235 (75.9)	1,986/8,235 (24.1)	1.02 [0.96–1.07]	0.579

N° of Past CD				
0	11,339/13,873 (81.7)	2534/13,873 (18.3)	Referent	Referent
1	263/1,285 (20.5)	1022/1,285 (79.5)	4.26 [4.14–4.38]	0.000
>1	1/97 (1.0)	96/97 (99.0)	5.26 [4.96–5.31]	0.000

N° of fetuses				
Singleton	11,573/15,025 (77.0)	3,452/15,025 (23.0)	Referent	Referent
Multiple	30/230 (13.0)	200/230 (87.0)	3.78 [3.57–3.95]	0.000

Presentation				
Cephalic	11,599/14,614 (79.4)	3,015/14,614 (20.6)	Referent	Referent
Abnormal lie	4/641 (0.6)	637/641 (99.4)	4.83 [4.79–4.84]	0.000

Birth weight (grams)				
2500–4000	10,428/13,320 (78.3)	2,892/13,320 (21.7)	Referent	Referent
<1000	12/52 (23.1)	40/52 (76.9)	3.25 [2.70–3.64]	0.000
1000–1499	10/84 (11.9)	74/84 (88.1)	3.76 [3.41–3.97]	0.000
1500–2499	329/723 (45.5)	394/723 (54.5)	2.41 [2.25–2.56]	0.000
>4000	824/1,076 (76.6)	252/1,076 (23.4)	1.07 [0.96–1.19]	0.218

10-Groups Variable				
1.Nlp, single ceph, ≥37 wks, spont lab	5,003/5,516 (90.7)	513/5,516 (9.3)	Referent	Referent
2.Nlp, single ceph, ≥37 wks, ind or pre-lab CD	977/1,703 (57.4)	726/1,703 (43.6)	3.08 [2.94–3.21]	0.000
3.Mlp*, single ceph, ≥37 wks, spont lab	4,362/4,474 (97.5)	112/4,474 (2.5)	0.29 [0.24–0.36]	0.000
4.Mlp*, single ceph, ≥37 wks, ind or pre-lab CD	537/708 (76.0)	171/708 (24.0)	2.10 [1.88–2.33]	0.000
5.Mlp, past CD, single ceph, ≥37 wks	245/1,199 (20.4)	954/1,199 (79.6)	4.23 [4.16–4.29]	0.000
6.7.9.All abnormal lie/breeches (past CD included)	4/641 (0.6)	637/641 (99.4)	4.63 [4.61–4.63]	0.000
8.Multiple pregnancies (past CD included)	30/230 (13.0)	200/230 (87.0)	4.39 [4.28–4.47]	0.000
10.Preterm, single ceph,<37 wks (past CD included)	445/784 (56.8)	339/784 (43.2)	3.10 [2.93–3.27]	0.000

Data are expressed in number, percentage and crude Risk Ratios – 95% confidence interval (bivariate analysis).

RR, risk ratios; CI, confidence interval; BMI, body mass index; CD, caesarean delivery; ceph, cephalic; Nlp, nulliparous; Mlp, multiparous; spont, spontaneous; ind, induced; lab; labor; wks, weeks.

* No past caesarean delivery.

p<0.05 is considered statistically significant.

### Multivariate analysis

**Figure 2:**
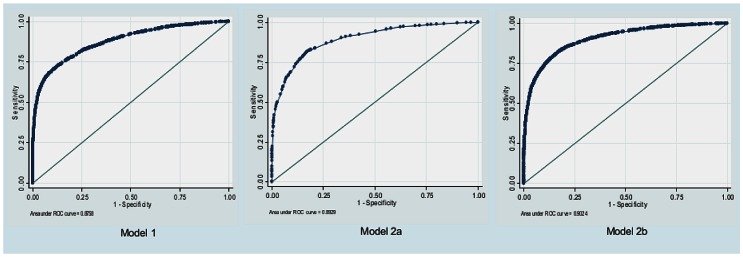
Receiver Operating Characteristic (ROC) curves for the multivariate logistic regression models. Footnotes: Model 1: Adjusted for maternal characteristics, pregnancy related variables and risk category. Model 2a: Adjusted for ten-groups. Model 2b: Adjusted for ten-groups, maternal characteristics and risk category.

Multiple logistic regression analyses modified substantially the inter-institutional variations of CD rates. In model 1 (adjusted for maternal characteristics, pregnancy related variables and risk factors), the CD rate of center F was not significantly different from the rate of center A, whereas rates of the other centers remained significantly higher than the referent. In model 2a (adjusted only for TGCS), CD rates of centers F and H were not significantly different from center A (Table 3). The two models showed different AUCs: 0.8758 and 0.8929 respectively for model 1 and 2a. The multivariate analysis considering TGCS and maternal characteristics did not modify significantly the results of model 2a (AUC: 0.8977). The evaluation including the TGCS associated with maternal age/BMI and risk factors (model 2b) showed a further reduction of CD rates variations (CD rates of centers F, H, I similar to referent) with the best performance of the test. The AUC of this model (0.9024) was statistically better than AUCs of both model 1 and 2a (Figure 2, Tables 3–4).

**Table 3:** Inter-institutional crude and adjusted Risk Ratios (RR, 95% Confidence Interval) for caesarean risk-adjustment models.

Center	No adjustment		Model 1		Model 2a		Model 2b	
	Crude RR	p-Value	Adj RR	p-Value	Adj RR	p-Value	Adj RR	p-Value
**A**	Referent	Referent	Referent	Referent	Referent	Referent	Referent	Referent
**B**	1.87 [1.65–2.09]*	0.000	2.00 [1.72–2.30]*	0.000	2.01 [1.69–2.34]*	0.000	1.98 [1.69–2.28]*	0.000
**C**	1.74 [1.52–1.98]*	0.000	1.89 [1.59–2.20]*	0.000	1.82 [1.50–2.17]*	0.000	1.83 [1.52–2.15]*	0.000
**D**	1.83 [1.64–2.02]*	0.000	1.95 [1.69–2.21]*	0.000	1.97 [1.69–2.26]*	0.000	1.93 [1.67–2.20]*	0.000
**E**	1.29 [1.11–1.48]*	0.001	1.32 [1.08–1.58]*	0.007	1.33 [1.08–1.62]*	0.007	1.34 [1.10–1.61]*	0.005
**F**	1.18 [1.01–1.36]*	0.033	1.18 [0.96–1.43]	0.104	1.16 [0.94–1.41]	0.170	1.19 [0.98–1.44]	0.097
**G**	1.38 [1.18–1.59]*	0.000	1.53 [1.25–1.83]*	0.000	1.35 [1.09–1.66]*	0.007	1.36 [1.10–1.65]*	0.005
**H**	1.23 [1.04–1.45]*	0.017	1.41 [1.14–1.71]*	0.002	1.10 [0.86–1.38]	0.452	1.19 [0.93–1.47]	0.153
**I**	1.44 [1.28–1.62]*	0.000	1.21 [1.01–1.43]*	0.043	1.29 [1.06–1.52]*	0.009	1.18 [0.98–1.41]	0.073
**L**	1.71 [1.47–1.98]*	0.000	1.98 [1.65–2.30]*	0.000	2.12 [1.75–2.50]*	0.000	2.13 [1.79–2.47]*	0.000
**M**	2.08 [1.89–2.26]*	0.000	2.16 [1.91–2.40]*	0.000	2.07 [1.80–2.34]*	0.000	2.00 [1.76–2.26]*	0.000

Center A is considered as the referent.

RR, risk ratios; CI, confidence interval.

Model 1: Adjusted for maternal characteristics, pregnancy related variables and risk category.

Model 2a: Adjusted for ten-groups.

Model 2b: Adjusted for ten-groups, maternal characteristics and risk category.

* p<0.05 is considered statistically significant.

**Table 4:** Assessment of the fit of risk-adjustment models.

	N° of observations	ROC Area	Standard Error	95% confidence Interval
**Model 1**	15,255	0.8758*	0.0036	0.86875–0.88283
**Model 2a**	15,255	0.8929**	0.0032	0.88667–0.89924
**Model 2b**	15,255	0.9024^†^	0.0031	0.89479–0.90691

Model 1: Adjusted for maternal characteristics, pregnancy related variables and risk category.

Model 2a: Adjusted for ten-groups.

Model 2b: Adjusted for ten- groups, maternal characteristics and risk category.

Chi Square  = 305.77, p = 0.00000.

* ** ^†^ p = 0.0000 for all pairwise comparisons (Bonferroni correction applied).

## Discussion

Rising CD rates are a cause of concern worldwide and risk adjustment (case-mix) analysis is recommended to assess CD rates as quality of health care among institutions [13]–[17], [23].

The aim of our study was to evaluate whether the TGCS could be considered as a reliable method for adjusting CD rates. Thus, we firstly evaluated CD rates adjusted for prospectively collected risk factors and customary maternal-obstetric variables. This preliminary step was essential to obtain a reference.

Looking at inter-institutional variations of CD rates, we considered center A as the referent center, whose rate appeared to be quite close to the recommended WHO rate [9].

After adjusting CD rates for the potential “customary” predictors and TGCS, we observed that both models differed from the crude analysis, reducing the inter-institutional variation of CD rates and supporting the observation that the analysis of crude RRs would have been misleading.

The association of TCGS with maternal characteristics and antenatal risk factors resulted as the most reliable model for CD case-mix adjustment. Our results are similar to those of Colais et al. However their results might be biased by the fact that they did not include maternal BMI. Furthermore it is unclear if they considered dystocia and fetal distress, a potential source of CD variation, into the risk adjustment evaluation [20].

Understanding the strengths and weaknesses of each model is an important step in trying to establish whether the TGCS can be applied in risk adjustment of CD rates.

The differences of institutional CD rates adjusted according to the two different models can be explained mainly by the different combination of variables: gestational age, parity, presence of past CD, single or multiple pregnancy and fetal presentation were considered separately in “customary” model 1, whereas the TGCS differentiated gestational age (<37 weeks or ≥37 weeks) according to fetal cephalic presentation and included either the presence of past CD in preterm deliveries/multiple pregnancies or in cases with abnormal fetal lie/presentation. Erroneously, we would expect the “customary” analysis of model 1, not dealing with a combination of variables, to be more efficient than the TGCS risk adjusted model. The better performance of the TGCS risk adjusted model, with or without the association of maternal characteristics and obstetrical risk factors, could be explained by the intrinsic principle of this classification system that allows prospective and reproducible identification of mutually exclusive and totally inclusive obstetric groups [19].

Our results should be interpreted considering some limitations. Firstly, we did not consider separately every antenatal risk factor for CD, labeling the pregnancy as “at risk” according to selected groups of risk conditions.

Previous studies adopted this classification considering that a successful model for adjusting CD rates should consider the most relevant risk factors and must be acceptable to practicing obstetricians. Moreover the evaluation of AUCs for each of the proposed risk models demonstrated consistently good predictive ability. Secondly, we did not consider in the risk adjustment other socio-demographic variables such as race/ethnicity and formal education. These variables, however, were not included because of the very low prevalence of non-Caucasians in our region and considering these two variables should not have a relevant role in the prediction of CD [13], [18], [24], [25].

Theoretically the inclusion of cases with induction of labor or pre-labor CD (groups 2 and 4) in the TGCS multivariate analyses might be questionable because it might be a source of increased CD rate among institutions and could justify hospitals with higher caesarean rates for doing more unnecessary inductions or elective caesarean sections. However, following this concern, we assessed the indications reported by clinicians for justifying inductions of labor or pre-labor CDs and these were appropriate in 99.6% of the cases.

Similarly to the study of Bragg et al., dealing with the variation in rates of caesarean section among English NHS [23], we decided to focus our attention on overall CD rates because of their clinical relevance. In this evaluation we considered the previous CD as a factor included both in the “customary” and in the TGCS adjusted models. This variable should be considered as an effect measure modifier in cases in which CD risk in women with previous CD is homogeneously distributed across centers. However, a) this is not our case as 7 out of 10 centers have significantly higher risks of CD if compared to the referent center, and b) in any case, “previous CD” is always strongly and independently associated with the risk of CD and should thus be included in case-mix adjusted models.

Every year, the Italian NHS publishes the results of the Outcome Evaluation Program (PNE) on risk adjusted “primary” CD rates [26]. The PNE website contains detailed documentation on the statistical procedures, the risk adjustment model and its impact on adjustment. However, the PNE reports no evidence on whether the TGCS should be considered as a reliable method for adjusting CD rates. Finally, even though the PNE has the advantage of being based on national data, it is built on routinely collected data which for their nature tend to be of suboptimal quality.

Even if our study was not based on a large number of deliveries, it should be considered one of the few in which CD rates were adjusted for unambiguous data, representing a reliable picture of the regional cohort. Information on maternal characteristic and antenatal obstetric conditions was prospectively gathered in a dedicated database and allowed us to collect standardized and homogeneous data, excluding only 3% of the records from the final analysis because of missing data. Medical records, birth certificates, diagnosis related group codes (DRG) and International Classification of Diseases – 9^th^ Revision (ICD-9) codes are commonly used as resource for research and quality surveillance in obstetric practice. However, it has been shown that they should be used with caution, given the high degree of variation in coding practices. Missing data or variability in data collection of obstetric and maternal characteristics might lead to potential bias and cesarean section rates might end up being adjusted by variables not homogeneously collected among institutions. Khan et al. observed that among 40,932 women with primary cesarean deliveries and no risk indicated on the birth certificate, 87% of cases had a risk identified in the hospital discharge data [27]. Chescheir et al. assessed the consistency of hospital coding for patients with cesarean delivery-related admissions among hospital coders from 11 institutions. They observed that consensus on DRG coding was found only in two thirds of cases and variation in use of ICD-9 codes existed with poor inter-institutional agreement [28]. Hanley et al., in their study based on British Columbia Perinatal Database Registry, did not adjust regional variations of cesarean section rates by past CD and BMI because of missing data on these variables in percentages ranging from 28% to 49% among institutions [29]. Colais et al. considered the TGCS and obstetrical risk factors into a risk adjusted CD model, extrapolating this data retrospectively from Hospital Discharge Abstracts (SDO) and Certificates of Delivery Care (CeDAP). On one side, they observed that one strength of their study was the opportunity to use two current administrative databases with a very good record linkage (higher than 95%). On the other side, they stressed that limitations of their results might be due to missing information on important risk factors and errors in coding, and that collecting information from administrative data may be biased by the basic conflict of interest that emerges from using the same data for reimbursement and for measuring quality [20].

In conclusion, our results support the belief that the evaluation of CD rates needs to be adjusted for complete and standardized potential predictors. In this context, the TGCS risk adjustment either with or without the inclusion of maternal characteristics and obstetrical risk factors, as predictors, might be considered as a reliable method to properly assess inter-institutional variation of CD rates. The main advantage of the TCGS risk adjustment model is represented by the possibility of conducting further analyses on mutually excludable subgroups, allowing for more detailed comparisons among institutions [11], [30].

## Supporting Information

Table S1 **Indications of induction of labor.** Footnotes: * pre-existing or gestational diabetes, pre-existing maternal disease suggesting the termination of pregnancy, obstetric cholestasis, alloimmunization, severe oligohydramnios, intrauterine growth restriction.
(DOC)

Table S2 **Indications of caesarean delivery.** Footnotes: *HIV, pre-existing or gestational diabetes, pre-existing maternal disease suggesting the termination of pregnancy, obstetric cholestasis, alloimmunization, severe oligohydramnios, intrauterine growth restriction.
(DOC)
