# Supplementary material for: The Application of the Ten Group Classification System (TGCS) in Caesarean Delivery Case Mix Adjustment. A Multicenter Prospective Study
Source: PLoS One. 2013 Jun 5;8(6):e62364. doi: 10.1371/journal.pone.0062364 (PMC3674002; doi:10.1371/journal.pone.0062364)
Supplement: Table S1 — Indications of induction of labor. Footnotes: * pre-existing or gestational diabetes, pre-existing maternal disease suggesting the termination of pregnancy, obstetric cholestasis, alloimmunization, severe oligohydramnios, intrauterine growth restriction. (DOC) [file pone.0062364.s001.doc]

**SUPPORTING INFORMATION**

Table S1. Indications of induction of labor.

| 1. Pre-labor rupture of membranes |
| --- |
| 1. Post term (gestational age≥ 41 weeks) |
| 1. Hypertensive disorders |
| 1. Other maternal reasons, e.g. procedure done for the benefit of the mother* |
| 1. Fetal reasons, e.g. procedure done for the benefit of the fetus* |
| 1. No absolute indications or no indication reported |

* pre-existing or gestational diabetes, pre-existing maternal disease suggesting the termination of pregnancy, obstetric cholestasis, alloimmunization, severe oligohydramnios, intrauterine growth restriction.
