# Supplementary material for: The Application of the Ten Group Classification System (TGCS) in Caesarean Delivery Case Mix Adjustment. A Multicenter Prospective Study
Source: PLoS One. 2013 Jun 5;8(6):e62364. doi: 10.1371/journal.pone.0062364 (PMC3674002; doi:10.1371/journal.pone.0062364)
Supplement: Table S2 — Indications of caesarean delivery. Footnotes: *HIV, pre-existing or gestational diabetes, pre-existing maternal disease suggesting the termination of pregnancy, obstetric cholestasis, alloimmunization, severe oligohydramnios, intrauterine growth restriction. (DOC) [file pone.0062364.s002.doc]

**SUPPORTING INFORMATION**

Table S2. Indications of caesarean delivery.

| 1. Suspicious or pathological cardiotocography |
| --- |
| 1. Other fetal reasons, e.g. procedure done for the benefit of the fetus* |
| 1. Other maternal reasons, e.g. procedure done for the benefit of the mother* |
| 1. Antepartal hemorrhage or placenta previa |
| 1. Preeclampsia or HELLP syndrome |
| 1. Breech presentation |
| 1. One previous caesarean delivery |
| 1. More than one caesarean delivery |
| 1. Dystocia-failed induction |
| 1. Dystocia- failure to progress |
| 1. No indication reported including maternal request |

*HIV, pre-existing or gestational diabetes, pre-existing maternal disease suggesting the termination of pregnancy, obstetric cholestasis, alloimmunization, severe oligohydramnios, intrauterine growth restriction.
